# Rituximab for treatment-resistant schizophrenia and/or obsessive-compulsive disorder (OCD): functional connectivity and cytokines associated with symptomatic improvements

**DOI:** 10.1192/j.eurpsy.2023.1309

**Published:** 2023-07-19

**Authors:** M. B. Humble, D. Eklund, D. Fresnais, U. Hylén, S. Sigra, P. Thunberg, S. Bejerot

**Affiliations:** School of Medical Sciences, Örebro University, Örebro, Sweden

## Abstract

**Introduction:**

Immunological mechanisms may contribute to the causation of mental illness. Autoimmunity is most convincingly shown for anti-NMDA-R encephalitis and Pediatric Acute-Onset Neuropsychiatric Syndrome (PANS); disorders that overlap clinically with schizophrenia and OCD. Altered inflammatory cytokine production, glial activation and auto-antibodies have also been associated with schizophrenia and OCD. In these disorders, however, the treatment results with anti-inflammatory or immunomodulating drugs have hitherto been limited and inconsistent. Yet other targets within the immune system may still be effective and new options are warranted for treatment-resistant patients. Rituximab targets B-lymphocytes and is often used in autoimmune disorders such as rheumatoid arthritis, multiple sclerosis and anti-NMDA-R encephalitis.

**Objectives:**

We aimed to investigate whether rituximab is clinically effective, safe and tolerable as add-on therapy in markedly ill, treatment-resistant adult psychiatric patients with schizophrenia or OCD. We also wanted to identify putative mediating mechanisms in treatment responders, such as cytokine changes and functional connectivity (FC).

**Methods:**

In an open pilot study, adults (18-39 years) with treatment-resistant schizophrenia and/or OCD were included. They received an intravenous infusion of rituximab 1000 mg, once at baseline, in addition to their regular psychiatric medication and were followed for 1 year. The main outcome measures were the Positive and Negative Syndrome Scale (PANSS) or Yale-Brown Obsessive Compulsive Scale (Y-BOCS), the Clinical Global Impression-Improvement scale (CGI-I) and the Personal and Social Performance scale (PSP). Treatment response was defined as ≥ 40 % decrease in PANSS or ≥ 35 % decrease in Y-BOCS, and much improved according to CGI-I. Resting-state fMRI was applied at baseline and after 5 months. Plasma cytokines were measured at 0, 3 and 5 months. Cognitive tests and the recently developed PsychoNeuroinflammatory Related Signs and Symptoms Inventory (PNISSI) were used to identify and measure symptoms related to neuro-inflammation and cognitive function.

**Results:**

Nineteen patients were treated with rituximab. 3-5 months after treatment, 6/9 patients with schizophrenia and 1/10 with OCD responded. One schizophrenia patient continues with rituximab every 6 months and has reportedly done well for almost 3 years. No severe side effects were reported apart from recurrent abdominal pain in a schizophrenia patient and one case of post-COVID-19 syndrome. Significant changes of FC were detected in responders only and correlated with PSP changes.

**Conclusions:**

Aberrant B-cell activities may contribute to treatment-resistant schizophrenia and be amenable to treatment with rituximab. However, the results of this pilot study need confirmation in placebo-controlled trials.

**Disclosure of Interest:**

None Declared

